# The Impact of Modifiable Parenting Factors on the Screen Use of Children Five Years or Younger: A Systematic Review

**DOI:** 10.1007/s10567-025-00523-9

**Published:** 2025-05-01

**Authors:** Blake Pyne, Olifa Asmara, Alina Morawska

**Affiliations:** 1 Parenting and Family Support Centre, School of Psychology, The University of Queensland, 13 Upland Road, St Lucia, Qld 4072 Australia; 2 Australian Research Council Centre of Excellence for Children and Families Over the Life Course, Brisbane, Australia

**Keywords:** Parenting, Screen use, Pre-schoolers, Portable media, Self-efficacy

## Abstract

Most children under age 5 exceed recommended screen time guidelines, with lifelong implications for children’s psychosocial, cognitive, socio-emotional, and physiological outcomes. Socio-ecological models point towards the important contribution of parental knowledge, modelling, practices, self-efficacy and style on child screen use. This study aimed to determine the extent to which these parenting factors influence child screen use, as well as the moderating role of screen media type (i.e. fixed vs. portable) on the relationship between parenting factors and child screen use. A systematic literature search for peer-reviewed studies published in English from 1982 to 2023 was conducted and yielded 87 eligible studies. Greater parental modelling, mealtime viewing, and the use of screens as a babysitting, mood regulation or reinforcement tool, were associated with higher child screen use. Contrastingly, higher levels of parental self-efficacy, and the implementation of screen use rules were associated with lower child screen use. Interventions targeting these parenting factors pose as a promising avenue to reduce child screen use. An authoritative parenting style was associated with lower child screen use, while authoritarian and permissive styles were associated with higher child screen use. The evidence for parental knowledge and co-viewing and, a neglectful parenting style was inconsistent or inconclusive. Finally, no observable trends or patterns in the data regarding the role of screen media type emerged. Future research should look to address issues in the measurement of child screen use to ensure comprehensive and objective assessments.

**Supplementary Information:**

The online version contains supplementary material available at 10.1007/s10567-025-00523-9.

## Introduction

In modern society, preschool-aged children (i.e. children 5 years or younger) are invariably exposed to screen media devices (Ralph & Petrina, 2019). While screen media sources such as television (TV) and computers are still popular among pre-schoolers (Tena et al., 2019), the advent of portable, interactive screen media such as smartphones and tablets has broadened screen use possibilities (Hutton et al., 2020; Neumann, 2015). Access to these devices has unsurprisingly translated to increased levels of screen use in children (Paus-Hasebrink et al., 2019), with the vast majority of Australian children under the age of 5 exceeding recommended screen time guidelines (Brushe et al., 2023; Rhodes, 2017; Tooth et al., 2019). On average, Australian children under the age of 2 are exposed to 2 h of screen use per day, and children between the ages of 2 and 5 are exposed to 3 to 4 h per day (Rhodes, 2017). The discrepancy between the recommended and actual time spent on screen media devices indicates that parents may be unaware of the recommendations (Hinkley & McCann, 2018; Miguel-Berges et al., 2020), consider the recommendations unrealistic (Ferguson, 2018), or are generally unconcerned about the accrued screen use of their children (He et al., 2005). Moreover, many parents may ignore recommendations in the belief that their child’s screen use serves a positive function, such as education (Zimmerman et al., 2007). This is concerning when considering the inverse relationship between greater screen use and unfavourable developmental outcomes (Haughton et al., 2015).

Young children may be particularly vulnerable to the effects of screen use as they are in a period of critical physical, cognitive, and social development (Irwin et al., 2007; Willumsen & Bull, 2020), linked to heightened levels of neuroplasticity (Conway & Stifter, 2012; Kolb & Fantie, 2009). The effects of screen use on children have been thoroughly assessed within the literature, and subsequently compiled in many systematic reviews and meta-analyses. These reviews have found a relationship between increased screen use and various negative psychosocial, cognitive, socio-emotional and physiological outcomes (LeBlanc et al., 2012; Stiglic & Viner, 2019). These negative outcomes may be associated with a lack of physical activities, sleep quality, social interaction, and hands-on sensorimotor stimulation due to excessive media use (John et al., 2023; Muppalla et al., 2023). Notably, among older children and adolescents, there is some contention surrounding the benefits and harms relating to screen use (Morawska et al., 2023). However, among preschool-aged children there is no evidence suggesting any health-related benefits of screen use (LeBlanc et al., 2012; Stiglic & Viner, 2019). Morawska et al. (2023) note that the functions of screen use change at different developmental stages, potentially resulting in different effects. Where screen media devices may be potentially beneficial for adolescents, such as engaging with peers online, it is unlikely that the same benefit applies to pre-schoolers; emphasising the importance of considering developmentally relevant effects. Given the negative outcomes, an understanding of the contributing factors to child screen use is essential.

To understand the screen use behaviours of young children a systematic approach, considering various interrelated influences of their environment is critical (Morawska et al., 2023). Socio-ecological models (Bronfenbrenner, 1992; Bronfenbrenner & Morris, 2006) are fundamental to explaining how proximal factors (i.e. experiences children directly encounter, such as parenting factors), and distal factors (i.e. variables that inform the proximal environment, such as socio-economic status) of a child’s socio-ecological environment can predict and inform their health and associated health behaviours. These factors are hierarchically organised and interact within and across levels of the model (Jenkins et al., 2015; McArthur et al., 2022). As such, the prediction and eventual prevention of potentially harmful health behaviours, such as excessive screen use, generally requires an understanding of both distal and proximal factors (Lämmle et al., 2013). However, to design effective prevention and intervention strategies for health behaviours, focus must be placed on modifiable factors (Wight et al., 2016). It is likely that proximal factors, specifically parent–child relationships, are the most significant modifiable influence on child screen use (Pereira et al., 2021; Rhodes et al., 2020).

Existing literature points to parental knowledge, modelling, practices, self-efficacy and style as the most important parenting factors related to screen use (Morawska et al., 2023; Philips et al., 2014). For example, greater parental understanding and accuracy of the screen use recommendations is significantly associated with less child screen use (Goh et al., 2016). While less frequently studied, increased parental knowledge of the associated health implications has been associated with lower screen use in children (Akbayin et al., 2023). Similarly, parents have an instrumental role in modelling healthy behaviours to their children (Andrews et al., 2010). Five systematic reviews found that greater parental screen use is associated with higher levels of screen use in children (De Craemer et al., 2012; Duch et al., 2013; Kaur et al., 2019; Veldman et al., 2023; Xu et al., 2015).

Parenting practices are the context-specific actions and behaviours made by parents that influence child screen use (Jago et al., 2013a). These practices may be the implementation of rules limiting screen use, mealtime viewing restrictions, using screens as a behaviour management tool or parent–child co-viewing. The presence of child screen limitations (i.e. rules) set by parents concerning the time and content of screen use was associated with lower child screen use in two systematic reviews (Cillero & Jago, 2010; Kaur et al., 2019). However, five reviews reported inconsistent results (De Craemer et al., 2012; Duch et al., 2013; Jago et al., 2013a; Veldman et al., 2023; Xu et al., 2015). A systematic review conducted by Xu et al. (2015) found that when prohibited from using screens during meals or whilst eating, children had reduced levels of overall screen use. However, three prior reviews found no relationship between mealtime viewing and overall screen use (De Craemer et al., 2012; Jago et al., 2013a; Veldman et al., 2023). Similarly, while the review of Xu et al. (2015) found that the amount of time children spend watching screens with their parents (i.e. co-viewing) is positively related to screen use, this was not supported in two other reviews which reported no association (Jago et al., 2013a; Veldman et al., 2023).

Parents may also use screens to manage behaviour and to achieve child-rearing objectives (Beyens & Egermont, 2014; Nabi & Krcmar, 2016). For instance, parents’ use of screens as a reinforcement tool for desired or unwanted behaviour is associated with increased screen use in their children (Tang et al., 2018; Thompson et al., 2016). Likewise, other less explored strategies such as using screens to keep a child occupied (i.e. “babysitting”), and as a tool for child mood regulation (i.e. calming) have also been found to increase screen use (Bayens & Eggermont, 2014; Elias & Sulkin, 2019). The implementation of these parenting practices has a bi-directional relationship with parent self-efficacy, wherein greater success at implementing such practices results in greater self-efficacy and vice-versa (Halpin et al., 2021).

Parental self-efficacy describes parents’ subjective convictions and beliefs about their ability to perform parenting tasks successfully; in particular, whether they can effectively manage the screen use of their child (Jago et al., 2015). Higher overall parental self-efficacy has been consistently associated with lower screen use in children across systematic reviews (Kaur et al., 2019; Veldman et al., 2023; Xu et al., 2015).

Additional research has been conducted to examine the relationship between broader parenting styles and screen use in pre-schoolers. Parenting style is defined as a dispositional, trait-like feature of parenting categorised by different levels of warmth and demand, where warmth is characterised by parents’ responsiveness, acceptance or involvement and demand reflects their strictness and control over child behaviour (Langer et al., 2014). Baumrind’s (1966), authoritarian (i.e. low warmth and high demand), authoritative (i.e. high warmth and high demand) and permissive (i.e. high warmth and low demand) parenting styles have been subject to most exploration in the screen use literature however, some studies have also considered the neglectful (i.e. low warmth and low demand) style proposed by Maccoby and Martin (1983). An authoritative parenting style has been associated with reduced levels of screen use in adolescents (Van der Geest et al., 2017) and young children (Detnakarintra et al., 2020; Schary et al., 2012; Veldhuis et al., 2014). However, the effect of authoritarian, permissive and neglectful styles, has varied (Howe et al., 2017; Langer et al., 2014). To clarify the role of parenting styles, three previous systematic reviews have been conducted, each determining that parenting style was not related to child screen use (Jago et al., 2013a; Veldman et al., 2023; Xu et al., 2015).

### Synthesis of Previous Systematic Reviews

The outcomes of studies examining the impact of parenting factors on screen use in children suffer from a lack of consistency, likely reflecting variance in study design, measurement tools and participant demographics (Xu et al., 2015). Despite attempts to clarify the role of parenting factors on screen use, the results of existing systematic reviews are mixed. This is problematic as there has been a failure to reach a general consensus on the significance of each parenting factor. Such inconsistency is likely explained by their diverging aims, search strategies, inclusion criteria and date of publication.

Five reviews have investigated the wider correlates of screen use in children, briefly examining the role of parenting factors (Cillero & Jago, 2010; De Craemer et al., 2012; Duch et al., 2013; Kaur et al., 2019; Veldman et al., 2023). Additionally, one has focussed on assessing the role of parenting factors on both physical activity and screen use simultaneously (Xu et al., 2015). Given their broad scope and purpose, it is likely that their aims, search strategies and inclusion criteria, result in a failure to capture all existing studies related to the role of parenting factors on screen use. These reviews employ a broad search strategy that involves the use of general search terms; risking the oversight of more nuanced search outcomes related to parenting factors. For instance, Veldman et al. (2023) did not use search terms related to parenting. Despite being suitable in the context of their aims, the search string does not directly capture studies related to parenting factors, as true of other reviews (e.g. Cillero & Jago, 2010; De Craemer et al., 2012; Duch et al., 2013; Kaur et al., 2019). Without a specific focus on the effect of parenting factors on childhood screen use, existing reviews have generated mixed search outcomes and subsequently reported different effects. This may also explain why potentially important predictors of screen use such as parental knowledge, reinforcement, babysitting, and mood regulation have been previously unobserved within reviews. Though an additional systematic review by Jago et al. (2013a) focussed specifically on the role of parenting factors, their scope was narrow, considering parenting practices and styles. Furthermore, the growing popularity of portable devices in the last decade has coincided with the arrival of novel screen use literature, the relevance of some past studies to the current context may be questionable (e.g. Cillero & Jago, 2010; De Craemer et al., 2012; Duch et al., 2013; Jago et al., 2013a; Xu et al., 2015). As such, there is great need for a current, comprehensive synthesis of information.

### The Current Study

The screen use of children poses a major health and developmental issue, thus an understanding of important contributing influences such as parenting factors is essential. As such, the primary aim of the current study was to conduct a systematic review to determine the relative importance of modifiable parenting factors (Morawska et al., 2023) on the screen use of preschool-aged children. We expected that higher levels of parental screen use knowledge (H1a), higher parental self-efficacy (H1b), and the implementation of screen use rules (H1c) would predict less screen use in pre-school aged children. In contrast, we expected that higher levels of parental modelling (H2a), co-viewing (H2b), mealtime screen viewing (H2c) and screens used to “babysit” (H2d), regulate a child’s mood (H2e) or reinforce their behaviour (H2f), would be associated with higher screen use. Furthermore, in light of the disparate findings between parenting style and pre-schooler screen use, we aimed to determine the extent to which authoritarian, authoritative, permissive and neglectful parenting styles influence screen use. We expected that authoritative parenting styles would be associated with significantly lower levels of screen use in children (H3a) (Detnakarintra et al., 2020; Schary et al., 2012; Veldhuis et al., 2014) and that authoritarian (H3b), permissive (H3c) or neglectful (H3d) styles would not be associated with child screen use (Howe et al., 2017; Langer et al., 2014).

In general, these effects have largely been investigated in the context of fixed media such as TVs, computers, and gaming consoles. However, the advent of portable, interactive screen devices such as mobile phones, tablets and gaming devices has changed the landscape of screen viewing, offering children ubiquitous and perpetual access to screen devices (Byrne et al., 2021; Hutton et al., 2020). This has potential implications on the relationship between parenting factors and screen use, such that the results of studies measuring fixed media may not translate to more recent studies measuring portable media types. As such, we adopt an exploratory approach to examine the extent to which the effect of modifiable parenting factors on screen use is moderated by screen media type (fixed vs. portable).

## Methods

A systematic review was conducted to address the study aims in accordance with the Preferred Reporting Items for Systematic Reviews and Meta-Analyses (PRISMA) guidelines, and the protocol was prospectively registered (PROSPERO: CRD42023425065).

### Search Strategy

In June 2023, a systematic search of the literature across four databases was conducted: PsycINFO, PubMed, Cumulative Index of Nursing and Allied Health Literature (CINAHL) and SCOPUS. The searches had no date restriction and were limited to articles published in the English language. The search strategy was developed using a combination of the free-text words for ‘*child’* (population), ‘*parent’* (exposure) and ‘*screen use’* (outcome) (see supplementary Table 1). To obtain relevant outcomes in titles and abstracts, controlled vocabulary terms and Boolean operators were applied and modified to suit each database. In addition to database searches, manual searches of relevant existing systematic reviews were conducted to ensure comprehensive coverage of the literature.

### Study Selection

Study selection was based on predetermined eligibility criteria. Studies were included if (a) they were quantitative and examined the relationship of parenting factors (i.e. modifiable influences or parenting style) with child screen use, (b) the mean age of child participants was less than six, (c) studies were written in English and published in a peer-reviewed journal, (d) the study design was observational, a randomised controlled trial, cross-sectional, longitudinal or a pilot study and (e) studies reported *p* values.

The exclusion criteria were (a) studies that exclusively used child-self report for any exposure or outcome measure and (b) studies that exclusively used a population of children with a developmental or physical disability, or children with a chronic illness. Children with developmental and physical disabilities or a chronic illness were excluded as they have known differences in screen use behaviours compared to general populations (Healy et al., 2020; Must et al., 2023).

After conducting independent database searches, all citations were merged, and duplicate records were removed. Titles and abstracts were screened for relevance according to the eligibility criteria by BP. The screening process was managed using the Covidence software. The full texts of relevant articles were evaluated by BP and OA. The disagreements between the reviewers were resolved through discussion of the eligibility criteria. Where discussion was unsuccessful (*n* = 2), the final decision was referred to AM.

### Data Extraction

Of the studies meeting the final selection criteria, demographical and methodological data were extracted. This included study design, country, participants (i.e. sample size, age, range, mean age), the parenting factor(s) (i.e. type(s), assessment tool(s) used), screen use (i.e. measure or assessment tool used), media type (i.e. measuring fixed, portable or both) and the main findings. Statistical significance was inferred when *p* < 0.05. The data extraction was conducted independently by two reviewers (BP extracted every study and a random sample (25%) were replicated by OA). The disagreements between the reviewers (< 1%) were resolved through discussion.

### Quality Assessment

The risk of bias assessment was conducted independently by BP and OA, where the methodological quality of each article was appraised at the study level. The Mixed Method Appraisal Tool (MMAT; Hong et al., 2018), was engaged for its versatility in assessing studies of different designs. For quantitative non-randomised studies, the MMAT assesses whether (1) the participants were representative of the target population, (2) the measures used are appropriate for both the outcome and exposure variables, (3) there were complete outcome data, (4) potential confounders were accounted for and (5) if the exposure occurred as intended. Outcomes of each item were defined as “yes” when meeting the criteria, “no” when not meeting the criteria, and “can’t tell” where the information was not reported or was unclear. Studies were ascribed a rating of low (1–2), medium (3) or high (4–5), as used in Evangelio et al. (2022). The ratings were calculated as a sum of yes (1), can’t tell (0.5) and no (0) responses to the five items. Disagreements between the reviewers (23.6%) were resolved through discussion.

### Evidence Synthesis

Given methodological and clinical (i.e. differences in exposures and outcomes) heterogeneity and the inability to calculate effect sizes (due to poor reporting quality), we adopted a narrative synthesis approach. For example, when considering parental self-efficacy, many barriers prohibited a prospective meta-analysis such as variance in study design, different definitions of self-efficacy (e.g. parental self-efficacy to restrict or manage screen time, parental confidence to influence physical and sedentary behaviour, parental self-efficacy to raise children, etc.) and screen use, and a failure to report important statistical information like effect sizes, means and standard deviations. As used in Cillero and Jago (2010), a summary coding system was employed to review the state of the literature for each factor. The percentage values were calculated based on the number of effects supporting the expected association divided by the total number of effects. The percentage of effects supporting a relationship was coded as strong (S; 75% to 100%), moderate (M; 60% to 74%), unclear (U; 34 to 59%) or no association (NA; < 33%). The factors observed in fewer than three studies were coded as insufficient (IN).

## Results

**Fig. 1:**
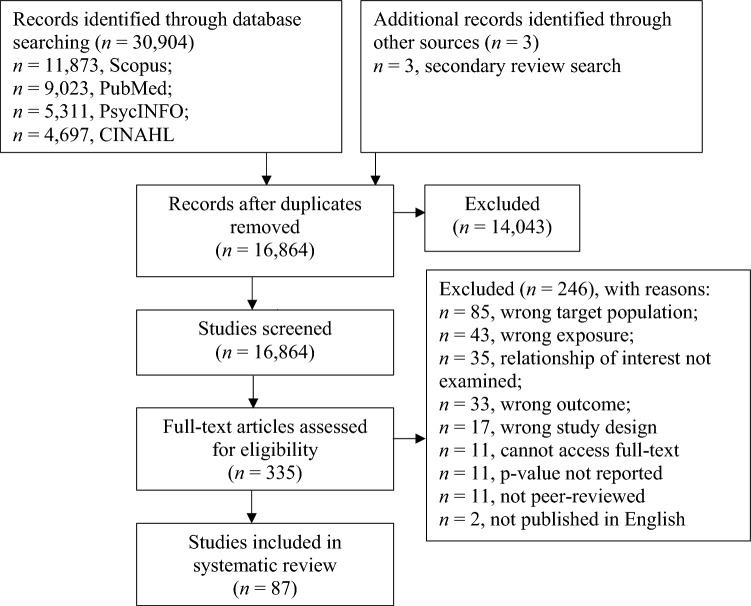
PRISMA flow diagram for study selection

The screening process is shown in Fig. 1. The primary database searches yielded a total of 30,904 articles. Three additional records were identified through a search of existing reviews. After removing duplicate articles (*n* = 14,043), 16,864 studies were screened by title and abstract. A total of 16,529 articles were considered irrelevant and therefore excluded. As a result, 335 studies were considered for full-text review and assessed for eligibility. A final total of 87 studies were included in this systematic review.

### Study Characteristics

The methodological and study characteristics of the included articles are presented in Table 1. Of the 87 articles, 31 originated from North America, 19 from Asia, 17 from Europe, 16 from Oceania and four from South America. The sample size of participants ranged from 45 to 10,967 across studies. Of those provided, the mean child age ranged from 2.0 to 5.8 years. The earliest study was conducted in 1982 and the most recent in 2023. Seventy-eight studies were cross-sectional, eight were longitudinal and one was a randomised controlled trial. Of the studies, 44.8% were published in the five years preceding the search date.

**Table 1:** Qualitative synthesis of methodological and study characteristics

ID	Study	Study design	Sample size	Country	Age Range (Years), Mean (SD)	Parenting factor(s) (Measure)	Child screen use (Measure)	Main findings
1	Abbott et al., 2016	Cross-sectional	450	Australia	3–5, 4.6 (0.7)	Parental Modelling (mother’s and father’s average TV viewing time)	Average TV viewing time (parent report). Reported separately for boys and girls	Higher mother TV viewing time predicted higher boys (*p* < .001) and girls (*p* < .001) viewing time. Fathers TV viewing time predicted boys (*p* < .05) and girls (*p* < .01) TV viewing time
2	Akbayin et al., 2023	Cross-sectional	486	France	0–6, 3.5 (1.5)	Parental Modelling (parent daily screen time: > 2 or < 2 h of screen use per day)	Average daily screen time (parent report)	Parents’ daily screen time was not associated with that of their children
3	Ali and Alma’aytah, 2022	Cross-sectional	2781	Jordan	0–5	1. Parental Knowledge (measure designed to assess Information parents possess about the advantages and disadvantages of smartphone exposure among young children in terms of their health) 2. Parental Modelling (parent self-reported average daily screen time hours)	Daily exposure to smartphones (parent report)	1. Greater parental knowledge was associated with lower smartphone exposure (*p* < .01) 2. Higher parental smartphone use was associated with higher child smartphone (*p* < .01)
4	Asplund et al., 2015	Cross-sectional	302	United States	0–5	1. Parental Modelling (parent daily screen time: > 2 h or < 2 h of screen use per day) 2. Parental Rules (parent reported TV restrictions)	Average daily screen time (parent report). Additionally, dichotomised as > 2 or < 2 h to reflect the American Academy of Paediatrics (AAP) screen recommendations	1. Parents using > 2 h of screens per day was associated with significantly greater overall child screen time (*p* < .001) 2. Parental TV restrictions were not associated with AAP guideline adherence
5	Barr et al., 2010	Cross-sectional	308	United States	0.5–1.5	Parental Rules (parent reported implementation of time restrictions)	24-h TV diary (parent report)	The implementation of time restrictions was not associated with TV use
6	Barr-Anderson et al., 2011	Cross-sectional	431	United States	5.8 (0.51)	1. Parental Modelling (average parental TV time, hours/day) 2. Parental Rules (parent reported implementation of limits around screen time)	Average daily screen time (parent report)	1. Parental TV time predicted higher average daily screen time of children (*p* < .001) 2. Greater implementation of limits around screen time were associated with reduced daily screen time of children (*p* < .01)
7	Bassul et al., 2021	Cross-sectional	332	Ireland	3–5, 4.37 (0.51)	1. Parental Modelling (parent reported average daily TV viewing time) 2. Parental Rules (parent reported implementation of screen time rules) 3. Mealtime Viewing (adapted from the Healthy Home Survey: Parents reported whether children were allowed to eat meals while watching TV)	Average daily screen time (parent report) Responses were dichotomised as > 1 or < 1 h to reflect the World Health Organisation’s (WHO) recommendations	1. Greater parental time watching TV was associated with children viewing > 1 h of screen time per day (*p* < .05) 2. The implementation of screen time rules was associated with children viewing > 1 h of screen time per day (*p* < .05) 3. Allowing meals to be eaten in front of the TV was associated with children viewing > 1 h of screen time per day (*p* < .001)
8	Bernard et al., 2017	Cohort Study	861	Singapore	2–3, T1: 2.03 (0.08) T2: 3.04 (0.09)	Maternal TV viewing times categorised as < 1 h, 1–2 h, 2–3 h or ≥ 3 h/day	Average daily screen time (parent report)	Maternal TV viewing times predicted higher daily screen time of children (*p* < .001)
9	Beyens & Eggermont, 2014	Cross-sectional	844	Belgium	0.5–6, 3.94 (1.23)	1. Parental Modelling (parental weekly TV viewing time, in hours) 2. Babysitting Tool (parents use of screens as a babysitter for their children. Measure adapted from Evans et al. (2011), Götz et al. (2007) and, Zimmerman et al. (2007) and assessed via four items)	Total weekly TV time, in hours (parent report)	1. Greater parental TV viewing was associated with higher levels of child weekly TV viewing time (*p* < .001) 2. The use of screens as a babysitting tool was associated with higher child screen time (*p* < .001)
10	Bleakley et al., 2013	Cross-sectional	465	United States	0–5	1. Parental Modelling (parent’s average daily TV viewing time) 2. Parental Rules (parent reported implementation of screen time rules) 3. Parental Co-Viewing (parent reported how often they watched TV with their child)	Average daily TV viewing (parent report)	1. Higher parent daily TV viewing time was associated with higher child TV viewing time (*p* < .05) 2. The implementation of screen time rules was not associated with child TV viewing time 3. Higher levels of parental co-viewing was associated with higher child TV viewing time (*p* < .05)
11	Carson & Janssen, 2012	Cross-sectional	746	Canada	0–5 3.0 (1.0)	1. Parental Modelling (parent’s average weekly screen time) 2. Parental Self-efficacy (parent’s confidence to reduce or eliminate their child’s screen time)	Average daily screen time (parent report)	1. Higher levels of parental screen time were associated with higher levels of child screen time (*p* < .001) 2. Higher levels of parental self-efficacy was associated with lower child screen time (*p* < .001)
12	Caylan et al., 2021	Cross-sectional	250	Turkey	2–5	Parenting Style (the parent attitude scale was used to determine alignment to authoritative, authoritarian, and permissive parenting styles)	Average daily screen time (parent report). Participants were then categorised as having low screen exposure (< 1 h average daily screen time) or high screen exposure (> 4 h of average daily screen time)	Children of parents with authoritative parenting styles were significantly more likely to be in the low exposure group, compared to the high exposure group (*p* < .001). Children of parents with authoritarian and permissive parenting styles were more likely to be in the high exposure group than in the low exposure group (*p* < .001)
13	Chen et al., 2020	Cross-sectional	4907	China	3–6 4.14	Parental Self-efficacy (parent self-perceived efficacy about their parenting skills. Adopted and modified from the Chinese parenting anxiety scale	Average daily screen time independently reported for TVs, tablets and computers (parent report)	Higher levels of parental self-efficacy were associated with lower levels of TV time (*p* < .001), tablet time (*p* < .001) and computer time (*p* < .05)
14	Chia et al., 2022	Cross-sectional	807	Singapore	2–5	Parental Modelling (parent’s average daily media use)	Average daily digital media use (parent report). Participants were then categorised as being in the bottom quartile or top quartile of screen viewers	Parent’s average daily media use was associated with increased child screen use on weekends (*p* < .01) and weekdays (*p* < .01)
15	Christakis et al., 2004	Cross-sectional	1454	United States	0–11, 5.05 (3.20)	Mealtime Viewing (parents reported whether children had eaten in front of the TV in the past week)	Average weekday media viewing time (parent report)	Eating meals in front of the TV was associated with higher levels of media viewing time (*p* < .05)
16	Cingel & Krcmar, 2013	Cross-sectional	168	United States	0.5–6, 2.62 (1.72)	1. Reinforcement Tool (parent reported use of media as a reward) 2. Mood Regulation Tool (parent reported use of screens to allow their child to relax)	Average daily screen exposure to TVs and computers (parent reported)	1. The use of screens as a reward was associated with increased TV and computer time (both *p* < .01) 2. The use of screens as a mood regulation tool was associated with increased TV and computer time (both *p* < . .01)
17	Corkin et al., 2021	Cross-sectional	3081	New Zealand	2, 2.07 (0.17)	1. Parental Rules (parent reported implementation of screen time rules) 2. Parental Co-Viewing (parent reported how often children are watching TV in the presence of an adult) 3. Parental Self-Efficacy (parents rated how confident they feel about being a parent)	Total screen time on the most recent weekday (parent reported)	1. The implementation of rules was associated with lower screen time in children (*p* < .001) 2. Co-viewing was associated with significantly lower screen time in children (*p* < .001) 3. Parental self-efficacy was not associated with screen time in children
18	Corkin et al., 2022	Longitudinal	5362	New Zealand	4–5, 4.54 (0.13)	1. Parental Rules (parent reported implementation of screen time rules) 2. Mealtime Viewing (parent reported frequency of the TV being on during meals)	Total screen time on an average weekday (parent reported)	1. The implementation of rules was associated with lower screen time in children (*p* < .05) 2. Children eating meals in front of the TV was associated with higher screen time in children (*p* < .001)
19	DeDecker et al., 2015	Cross-sectional	AU: 947 BE: 1527	Australia (AU) Belgium (BE)	AU: 4.5 (0.7) BE: 4.4 (0.6)	1. Parental Modelling (AU parents provided total weekly time. BE parents provided average daily screen time) 2. Parental Rules (parent reported implementation of screen time rules)	Total weekly TV viewing (parent reported)	1. The effect of parental TV time on the screen time of children was associated with increased screen time in children across AU and BE populations (both *p* < .001) 2. The implementation of rules was associated with lower levels of child screen time across AU and BE populations (both *p* < .001)
20	Detnakarintra et al., 2020	Longitudinal	280	Thailand	T1: 3 (0.03) T2: 4	Parenting Style (Assessed via the Parenting Styles and Dimensions Questionnaire‐short version. Strict authoritarian, nurturing authoritative and relaxed permissive outcomes were derived at T1 and T2)	24-h media diary. Collected at T1 and T2. (parent report)	At three years, higher scores on the nurturing authoritative parenting style measure was associated with lower child screen time (*p* < .01), however higher scores on the relaxed permissive parenting style or strict authoritarian parenting style measures had children with higher total screen time (*p* < .01). The same effects (and significance levels) were true of children at four years
21	Downing et al., 2015	Cross-sectional	935	Australia	3–5	Parental Rules (parent reported implementation of limits placed on TV, computer, and electronic game viewing)	Average daily screen time (parent reported)	In both boys and girls, the implementation of screen time rules was associated with lower child screen time (both *p* < .05)
22	Downing et al., 2017	Cross-sectional	937	Australia	3–5, 4.50 (0.70)	1. Parental Modelling (mother and father reported TV viewing (hours/week)) 2. Parental Self-Efficacy (parent reported self-efficacy to limit screen time	Average daily screen time (parent reported). Reported separately for boys and girls	1. In both boys and girls, maternal and paternal TV viewing was significantly associated with increases in screen time (all *p* < .05) 2. In both boys and girls, greater parental self-efficacy to limit screen time was associated with reductions in screen time (both *p* < .05)
23	Elias & Sulkin, 2019	Cross-sectional	289	Israel	1.5–3, 2.17 (0.57)	1. Mealtime Viewing (parent reported use of screen to facilitate mealtime) 2. Parental Co-Viewing (parent reported co-viewing of children’s and adult programmes) 3. Reinforcement Tool (parent reported use of screens as a reward for desirable behaviour) 4. Mood Regulation Tool (parent reported use of screens to calm their child) 5. Babysitting Tool (parent reported use of screens to keep their child occupied	Total screen time on an average weekday and weekend (parent reported)	1. Mealtime screen use was associated with higher levels of screen time in children on weekday’s and weekends (*p* < .01 and *p* < .05, respectively) 2. Co-viewing of child or adult related programmes was not associated with child screen time on weekday’s. Co-viewing child programmes likewise, did not predict weekend viewing. However, co-viewing adult programmes did predict weekend viewing (*p* < .01) 3. Rewarding children with screen viewing was associated with higher screen viewing in children on weekday’s and weekend’s (*p* < .05 and *p* < .01, respectively) 4. Parent’s using screens to calm their child was positively related to screen time on weekday’s and weekend’s (both *p* < .01) 5. Using screen to babysit was associated with higher levels of screen time (both *p* < .01)
24	Fitzpatrick et al., 2022	Cross-sectional	316	Canada	2–5, 3.45	1. Parental Modelling (parent reported average media viewing per day) 2. Parental Rules (parent reported implementation of rules and limits on child media activities) 3. Parental Co-Viewing (parent reported social co-viewing)	Average daily screen time (parent reported). As determined by the media assessment questionnaire Responses were dichotomised as > 2 or < 2 average daily screen time	1. Higher parental media viewing predicted children having > 2 h of screen time per day (*p* < .001) 2. The implementation of rules and limits predicted children not viewing > 2 h per day (*p* < .001) 3. Parental co-viewing did not predict children exceeding screen time guidelines
25	Frata et al., 2021	Cross-sectional	237	Brazil	3–5	Parental Modelling (parent reported total screen time exposure)	Total weekly screen time exposure (parent reported)	Higher parental screen time exposure was associated with higher overall screen time exposure in children (*p* < .001)
26	Gao et al., 2022	Cross-sectional	10,967	China	3–6, 4.82 (1.05)	Parental Modelling (mother and father reported parent daily screen time: > 2 h or < 2 h of screen use per day)	Average daily screen time (parent reported)	When both fathers and mothers had > 2 h screen time children had significantly higher screen time compared to children of fathers and mothers who had < 2 h screen time (both *p* < .01)
27	Goh et al., 2016	Cross-sectional	725	Singapore	0–2	1. Parental Knowledge (measure assessed knowledge of screen viewing recommendations for children < 2 by asking whether parents believed screen viewing should be minimised, should be < 2 h or > 2 h 2. Parental Modelling (parent reported total weekly screen viewing. Categorised into low, medium and high tertiles) 3. Parental Rules (parent reported implementation of rules on both time and program content)	Total weekly screen viewing (parent reported)	1. Knowledge of screen time viewing recommendations was not associated with child screen viewing 2. Parent reported screen time was not associated with screen viewing in children 3. Parental rules on program content were not significantly associated with child screen viewing. However, parental rules on time were associated with reduced child screen viewing (*p* < .001)
28	Goncalves et al., 2022	Cross-sectional	306	Brazil	3–6, 5.17 (0.92)	1. Parental Modelling (parent reported modelling and enjoyment of screen time. Measured using scales developed by Vaughn et al. (2013)) 2. Parental Rules (parent reported implementation of screen time limits. Measured using scales developed by Vaughn et al. (2013))	Average daily screen time (parent reported). Measured using items from the Australian InFANT study (Hesketh et al., 2013). Responses were dichotomised as > 1 or < 1 h to reflect the WHO recommendations	1. Parental modelling was associated with decreased odds of children engaging in < 1 h of screen time per day (*p* < .01) 2. The implementation of parental rules was associated with children meeting screen time guidelines (*p* < .05)
29	Guedes et al., 2020	Cross-sectional	244	Brazil	2–4, 2.96 (0.47)	1. Parental Rules (parent reported implementation of rules on both time and inappropriate content)	Total daily interactive media time. Children were categorised into three groups: group 1 – no screen time, group 2 – < 45 min per day, group 3 – < 45 min per day	1. parents setting limits on time and inappropriate content was not associated with group membership
30	Guo et al., 2022	Cross-sectional	867	Japan	0–6, Urban: 4.6 (0.9) Rural: 4.5 (0.9)	1. Parental Modelling (parent reported entertainment viewing habits. Measured using the SMALLQ® questionnaire developed by Chia et al. (2022)) 2. Parental rules (parent reported implementation on the amount of screen time) 3. Parental Co-Viewing (parent reported co-participation in children’s digital media and physical play. Measured using SMALLQ®)	Screen time was assessed as a function of parent reported use of screens for entertainment and communication. Independent results were calculated for urban and rural samples	1. Parental modelling was significantly associated with higher child viewing in urban and rural children (both *p* < .01) 2. Parent’s implementation of screen time rules was associated with less child screen use in the urban sample (*p* < .01), but not in the rural sample 3. Parent’s co-participation in screen time was not associated with children’s screen viewing in urban or rural samples
31	Halpin et al., 2021	Cross-sectional	107	Australia	0–4, 2.63 (1.27	Parental Self-Efficacy (parent reported self-efficacy was measured using the screen time self-efficacy scale	Average daily screen time (parent reported)	Higher parental self-efficacy was associated with lower child screen time (*p* < .001)
32	Hinkley et al., 2013	Cross-sectional	935	Australia	3–5, 4.54 (0.70)	1. Parental Modelling (mother and father self-reported time in TV/DVD/video viewing (hours)) 2. Parental Rules (parent reported implementation of limits on the amount of time child is allowed to watch TV) 3. Parental Self-Efficacy (parent reported confidence to restrict child’s time on computer/e-games)	Screen time was assessed as a function of parent reported use of screens for entertainment and communication. Responses were dichotomised as > 2 or < 2 h to reflect the AAP recommendations) where compliance to the guidelines was assessed	1. Maternal TV time did not predict boys’ and girls’ adherence to AAP guidelines (both *p* < .001). Paternal TV time did not predict guideline adherence 2. In girls, the implementation of screen time limits did not predict adherence AAP recommendations 3. In girls, parental confidence to limit computer/e-games was not associated with adherence to the AAP recommendations
33	Hnatiuk et al., 2015	Longitudinal	404	Australia	0.33–1.58	Parental Self-Efficacy (mother reported self-efficacy for limiting children’s screen viewing)	Total weekly screen time (parent reported)	Higher levels of maternal self-efficacy was associated with reduced levels of screen time in children (*p* < .001)
34	Holman & Braithwaite, 1982	Cross-sectional	282	Australia	3–6, 3.4	Parental Modelling (parent reported importance of TV)	Total weekly screen time (parent reported)	High leisure TV time in mothers and fathers was associated with increased screen time in children (both *p* < .001)
35	Howe et al., 2017	Cross-sectional	487	New Zealand	2	Parenting Style (assessed mothers and fathers via a 30-item self-report questionnaire which provided scores on authoritarian, authoritative and permissive parenting)	Average daily screen time (parent reported)	In mothers, an authoritative parenting style was associated with lower levels of screen time in children (*p* < .05). Authoritarian and permissive styles were associated with increased screen time in children (*p* < .01 and *p* < .001, respectively). In fathers, an authoritative parenting style was not associated with screen time in children. However, authoritarian and permissive styles were associated with increased screen time (*p* < .01 and *p* < .05, respectively)
36	Howie et al., 2020	Cross-sectional	96	Australia/ United States	0–5, 3.00 (1.10)	Parental Modelling (using the technology use questionnaire (TechU-Q) parents self-reported their total use of TVs, Tablets and Mobiles)	Using the TechU-Q parents reported their child’s total use of TVs, Tablets and Mobiles	Parent’s TV, tablet and mobile use was associated with higher levels of child use of each device (*p* < .001, *p* < .01, *p* < .01, respectively)
37	Huang et al., 2020	Cross-sectional	699	China	2–7, 4.76 (0.96)	Parenting Style (parents completed the questionnaire of parenting styles to attain outcomes of authoritarian, permissive, uninvolved (neglectful) and democratic (authoritative) parenting styles)	Average video game use frequency (parent reported)	Permissive, neglectful and authoritarian parenting styles were associated with higher frequencies of video game use in children (*p* < .01, *p* < .01 and *p* < .05, respectively). However, an authoritative parenting style was associated with less video game use (*p* < .05)
38	Jago et al., 2013b	Cross-sectional	252	England	2.3–5.6, 3.7	1. Parental Modelling (parent reported average daily screen time. Responses were dichotomised as (> 2 h or < 2 h per day) 2. Parental Self-Efficacy (parent reported self-efficacy to limit the screen viewing of their children)	Average daily screen time (parent reported) Responses were dichotomised as > 2 or < 2 h per day	1. Children of parents who had over > 2 h of screen time per day were more likely to have > 2 h of screen time per day themselves (*p* < .01) 2. Higher parental self-efficacy was associated with children not viewing > 2 h of screen time per day (*p* < .01)
39	Jiang et al., 2006	Cross-sectional	930	China	2–6, 4.6 (1.1)	Parental Modelling (mother and father reported daily TV viewing hours)	Daily TV viewing hours (parent reported)	Both mother’s and father’s daily TV viewing hours were associated with higher child daily TV viewing hours (both *p* < .05)
40	John et al., 2021	Cross-sectional	189	India	2–5	1. Parental Rules (parent reported implementation of screen time limits) 2. Mealtime viewing (parent reported child mealtime screen use) 3. Co-viewing (parent reported shared screen use with their child)	Average daily screen time (parent reported). Participants were grouped according to whether they had > 1 or < 1 h of screen time per day	1. The implementation of screen time limits by parents was associated with children having > 1 h of screen use per day (*p* < .05) 2. Mealtime screen viewing in children was associated with children having > 1 h of screen use per day (*p* < .05) 3. Co-viewing was not associated with child group membership
41	Jusiene et al., 2019	Cross-sectional	847	Lithuania	2–5, 3.79 (1.15)	Mealtime Viewing (parent reported frequency of screen use during meals)	Average daily screen time (parent reported)	Higher levels of screen use during meals were associated with higher overall screen time in children (*p* < .01)
42	Kaur et al., 2022	Cross-sectional	360	India	2–5, 3.5 (0.9)	1. Parental Modelling (parent self-reported average daily screen time. Responses dichotomised as > 2 or < 2 h per day) 2. Parental Rules (parent reported implementation of screen time rules)	Average daily screen time (parent reported) Responses were dichotomised as > 1 or < 1 h per day	1. Children of parent’s with > 2 h of screen time per day were more likely to exceed > 1 h of screen time per day (*p* < .05) 2. The presence of screen time rules did not predict children exceeding the screen time guidelines
43	Kennedy, 2000	Cross-sectional	45	United States	4–5	Parental Co-Viewing (parent reported co-viewing with their children)	Average daily screen time (parent reported)	Parental co-viewing was associated with higher child screen time (*p* < .001)
44	Kieslinger et al., 2021	Randomised Controlled Trial	558	Germany	3–5, 3.6 (0.6)	Parental Self-Efficacy (parent self-reported parenting self-efficacy)	Average daily screen time (parent reported)	Parental self-efficacy was associated with increased child screen time (*p* < .01)
45	Konok et al., 2020	Cross-sectional	96	Hungary	0–7, 3.00 (1.85)	Parenting Style (parents responded to the digital kids questionnaire which provided outcomes for authoritarian, authoritative and permissive parenting styles)	Average daily mobile touch screen device use (MTSD) (parent reported)	Children of parents with a permissive or authoritative parenting style had higher MSTD use (both *p* < .05), whereas children of parents with an authoritarian parenting style had lower MSTD use (*p* < .001)
46	Kourlaba et al., 2009	Cross-sectional	2374	Greece	1–5	Parental Modelling (mother and father average daily TV viewing time)	Average daily TV viewing time (parent reported). In addition to the continuous measure, responses were dichotomised as > 2 or < 2 h per day to reflect the AAP screen time recommendations	In children aged 1–2 years, mother’s and father’s TV viewing was associated with higher child screen time (both *p* < .05). However, neither mother’s or father’s TV viewing predicted children viewing > 2 h of screen time per day. In children aged 3–5 years, mother and father TV viewing was associated with higher child screen time (both *p* < .05). Both mother’s and father’s TV viewing time predicted children viewing > 2 h per day (both *p* < .05)
47	Lammers et al., 2022	Cross-sectional	178	United States	0–2, 1.04 (0.55)	1. Parental Knowledge (parent reported awareness of the AAP screen time guidelines) 2. Reinforcement Tool (parent reported use of screen time to reward their child for good behaviour) 3. Mood Regulation Tool (parent reported use of screen time to help their child calm down or relax)	Average daily screen time (parent reported)	1. Mothers who were aware of the screen time guidelines had children with less screen time compared to mothers who were unaware of the screen time guidelines (*p* < .05) 2. Parents use of screens as a reward for good behaviour was not associated with child screen time 3. Parents use of screens to help their child calm down or relax was not associated with child screen time
48	Lampard et al., 2013a	Cross-sectional	147	United States	2–6, 3.7 (0.9)	Parental Rules (parent reported restriction of screen time)	Average daily screen time (parent reported)	Greater parent restriction of child screen time was associated with lower overall levels of screen time in children (*p* < .001)
49	Lampard et al., 2013b	Cross-sectional	146	United States	2–5	Parental Rules (parent reported adherence to their children having < 2 h of screen use per day. Responses were converted to a dichotomous variable where parents were categorised as “high screen time restrictors” or “low screen time restrictors.”	Average daily screen time (parent reported). Responses were dichotomised as > 1 or < 1 h per day, following WHO recommendations	Children of high restrictors were more likely to meet the WHO recommendations compared to children of low restrictors (*p* < .01)
50	Lauricella et al., 2015	Cross-sectional	2300	United States	0–8, 4.20 (2.58)	Parental Modelling (parent reported average daily screen time)	Average daily screen time on TVs, computers, smartphones and tablets (parent reported)	Greater parental screen time was associated with higher child TV, computer, smartphone and tablet use (all *p* < .001)
51	Lee et al., 2021	Cross-sectional	CA: 121 KR: 101	Canada (CA) South Korea (KR)	2–5, CA: 3.4 (0.7) KR: 3.4 (1.2)	1. Parental Modelling (parent reported average screen time modelling per day) 2. Parental Self-Efficacy (parent reported barrier self-efficacy for limiting screen time)	Average daily screen time (parent reported)	1. Higher parental modelling was associated with higher screen time in children (*p* < .001) 2. Higher barrier self-efficacy was associated with lower screen time in children (*p* < .001)
52	Lee et al., 2018	Cross-sectional	193	Canada	1.6 (0.2)	Parental Self-Efficacy (parent reported barrier self-efficacy for limiting screen time)	Average daily screen time (parent reported)	Higher barrier self-efficacy was associated with lower levels of screen time in children (*p* < .01)
53	Lee et al., 2009	Longitudinal	2902	United States	0–4	Parental Rules (parent reported implementation of screen time limits)	24-h TV diary (parent report)	The implementation of parental rules was associated with lower levels of child screen time (*p* < .01)
54	Levine et al., 2019	Cross-sectional	326	United States	0–3, 1.43 (0.83)	1. Parental Modelling (parent self-reported frequency of mobile media use) 2. Parental Co-Viewing (parent reported frequency of children using mobile media with a parent)	Frequency of child mobile media use	1. Parent mobile media use was associated with greater child mobile media use (*p* < .001) 2. Child mobile media use with a parent was associated with greater overall child mobile media use (*p* < .001)
55	Lusted & Joffe, 2018	Cross-sectional	162	Australia	4.96 (1.21)	Parental Modelling (parent self-reported weekly viewing time)	Weekly viewing time (parent reported)	Greater parental weekly viewing time was associated with lower levels of screen time in children (*p* < .01)
56	Määttä et al., 2017	Cross-sectional	864	Finland	3–6, 4.70 (0.89)	1. Parental Modelling (parent reported average daily screen time (for leisure) in front of children) 2. Parental Rules (parent reported implementation of screen time rules) 3. Parental Self-Efficacy (parent reported self-efficacy for limiting children’s screen time)	Average daily screen time (parent reported)	1. Higher levels of parental screen time were associated with higher levels of child screen time (*p* < .001) 2. The implementation of screen time rules was associated with lower child screen time levels (*p* < .001) 3. Higher levels of parental self-efficacy was associated with higher levels of child screen time (*p* < .001)
57	Morowatisharifabad et al., 2015	Cross-sectional	188	Iran	3–5, 4.13 (0.74)	Parental Modelling (mothers and fathers self-reported average daily screen time)	Average daily screen time (parent reported)	Fathers average daily screen time was not associated with higher child screen time. Mothers average daily screen time was associated with higher child screen time (*p* < .05)
58	Nabi & Krcmar, 2016	Cross-sectional	151	United States	0–5-6, 2.87 (1.61)	1. Reinforcement Tool (parent reported use of screens as a reward) 2. Mood Regulation Tool (parent reported use of screens to help their child relax) 3. Babysitting Tool (parent reported use of screens to get time to themselves)	Average daily screen exposure (parent reported)	1. Parents use of screens to reward children was associated with higher levels of child screen time (*p* < .001) 2. Parents use of screens to relax their child was associated with higher child screen time (*p* < .001) 3. Parents use of screens to get time to themselves was associated with greater child screen stime (*p* < .001)
59	Neshteruk et al., 2021	Longitudinal	252	United States	2–5	1. Parental Modelling (parent reported explicit modelling/enjoyment of screen time) 2. Parental Rules (parent reported limiting of screen time) 3. Reinforcement Tool (parent reported use of screen time to reward/control behaviour)	Total weekly screen time (parent reported)	1. Parent modelling of screen time was not associated with child screen time 2. Limiting screen time was not associated with child screen time 3. The use of screen time as a reward or to control behaviour was not associated with child screen time
60	Nevski & Silbak, 2016	Cross-sectional	400	Estonia	0–3	1. Parental Modelling (parent reported frequency of screen device use) 2. Parental Rules (parent reported implementation of rules that restrict, location, content and time on screens)	Frequency of touchscreen device use (parent reported)	1. Greater frequency of parental screen device use was associated with greater frequency of child screen device use (*p* < .05) 2. Parent reported implementation of viewing limits was associated with children using smart devices more frequently (*p* < .05)
61	Nikken & Schols, 2015	Cross-sectional	896	Netherlands	0–7, 3.42 (2.27)	1. Parental Modelling (parent self-reported average daily screen time) 2. Mood Regulation Tool (parent reported use of media as a pacifier)	Average daily screen time on TVs, game devices, computers, and touchscreens (parent reported)	1. When parents spend more time on electronic devices, children spent more time watching TVs, game devices, computers and touchscreens (*p* < .001, *p* < .001, *p* < .01, *p* < .01, respectively) 2. Screens used for the purpose of pacifying children were not associated with child TV, game device or computer use but did predict increased touchscreen use (*p* < .001)
62	Njoroge et al., 2013	Cross-sectional	596	United States	3–5	Parental Self-Efficacy (parent reported confidence to manage TV exposure of children)	1-week media diary (parent report)	Parental confidence to manage child TV exposure was associated with lower TV exposure in children (*p* < .05)
63	Oflu et al., 2021	Cross-sectional	240	Turkey	2–5	Parental Co-Viewing (parent reported co-viewing with children)	Average daily screen time (parent reported). Responses were dichotomised as < 1 h or > 4 h per day	When children did not co-view with their parents, they were more likely to engage in > 4 h of screen time (*p* < .05)
64	Pedrotti et al., 2022	Cross-sectional	Pre-COVID: 257 During-COVID: 256	Brazil	0–3, Pre-COVID: 1.95 (0.82) Post-COVID: 1.37 (0.85)	Parental Modelling (parent self-reported total weekly screen time)	Total weekly screen time (parent reported)	Parents media use was associated with higher child screen time prior to the COIVD-19 pandemic (*p* < .001). However, no association was reported during the pandemic
65	Rai et al., 2022	Cross-sectional	105	Canada	3–5, 4.5 (0.68)	Parental Modelling (parent reported average hors of screen time per day)	Total weekly screen time (parent reported)	Parent screen time was associated with higher child screen time (*p* < .05)
66	Raj et al., 2022	Cross-sectional	489	Malaysia	0–5	1. Parental Modelling (parent self-reported average daily screen time. Responses dichotomised as > 2 or < 2 h of screen time per day) 2. Parental Rules (parent reported implementation of screen related restrictive practices) 3. Parenting Style (parent self-reported responses to an involvement and strictness scale. Participants were categorised as authoritative or non-authoritative based on their responses)	Average daily screen time (parent reported)	1. Children of parents with > 2 h of screen time per day had higher screen time than children of parents < 2 h (*p* < .01) 2. The implementation of screen related restrictive practices were not associated with child screen time 3. An authoritative parenting style was not associated with child screen time
67	Schary et al., 2012	Cross-sectional	201	United States	2–5, 4.00 (1.30)	Parenting Style (parental warmth and control scores were calculated to obtain authoritative, authoritarian and permissive styles)	Average daily screen time on weekdays and weekends (parent reported)	On weekdays and weekends, and authoritative parenting style was associated with lower child screen time (*p* < .05 and *p* < .01, respectively) however, authoritarian and permissive styles were not associated with screen time
68	Sigmund et al., 2016	Cross-sectional	194	Czech Republic	4–7, Boys: 5.6 (0.9) Girls: 5.6 (0.9)	Parental Modelling (mother and father self-reported average daily screen time)	Average daily screen time (parent reported)	Greater mother and father screen time was associated with higher child screen time (both *p* < .001)
69	Smith et al., 2010	Cross-sectional	764	Australia	1.7–5.6, 3.90 (0.80)	Parental Self-Efficacy (parent reported confidence to influence child’s physical activity)	Average daily screen time (parent reported). Responses were dichotomised as > 2 or < 2 h of screen time	Greater self-efficacy predicted children exceeding screen use guidelines (i.e., > 2 h) (*p* < .05)
70	Tan et al., 2023	Cross-sectional	255	China	4.41 (0.8)	1. Parental Modelling (parent self-reported average daily screen time in the presence of their child) 2. Parent Co-Viewing (parent reported frequency of using devices with their children)	Average daily screen time on weekdays and weekends (parent reported)	1. On weekday’s parent’s screen time was not associated with their children’s screen time. However, on weekend’s, higher parental screen time was associated with greater child screen time (*p* < .05) 2. On both weekdays and weekends parental co-viewing with children was associated with higher screen time (both *p* < .001)
71	Tang et al., 2018	Cross-sectional	62	Canada	1.5–5, 3.65 (1.36)	1. Parental Modelling (mother and father reported modelling of screen use) 2. Parental Rules (parent reported implementation of screen time limits) 3. Mealtime Viewing (parent reported child use of screens during mealtime) 4. Reinforcement Tool (parent reported use of screens to control behaviour)	Average daily screen time on weekdays and weekends (parent reported)	1. On weekdays, mothers modelling of screen use was associated with greater screen time in children (*p* < .05), however no association was reported for fathers modelling. Likewise, on weekends, neither mothers nor fathers modelling of screen use was associated with child screen time 2. On both weekdays and weekends, both mothers and fathers implementation of screen time limits were associated with reduced child screen time (all *p* < .01) 3. On weekdays, when mothers and fathers allowed screen time during meals, children had higher screen time (both *p* < .05). However, on weekends no associations were present 4. On weekdays, mothers use of screens to control behaviour was associated with greater screen time (*p* < .05), however this was not true of fathers. On weekends both mothers and fathers use of screens to control behaviour was associated with increased screen time (*p* < .01 and *p* < .05, respectively)
72	Thompson et al., 2016	Cross-sectional	309	United States	3–5	1. Parental Modelling (parent self-reported average daily TV viewing) 2. Mealtime Viewing (parent reported frequency of mealtime screen viewing)	Background TV exposure	1. Parent’s average daily screen time was not associated with child background TV exposure 2. Increased frequency of mealtime screen viewing was associated with higher child exposure to background TV (p < .05)
73	Thompson et al., 2018	Cross-sectional	312	United States	3–5, 3.91 (0.79)	1. Parental Knowledge (parent reported knowledge related to child screen use) 2. Parental Rules (parent reported implementation of screen time restrictions) 3. Mealtime Viewing (parent reported child use of screens during meals) 4. Parental Self-Efficacy (parent reported self-efficacy to restrict child screen time)	Average daily screen time (parent reported via screen use diaries)	1. Greater parental knowledge related to child screen use was associated with lower levels of child screen time (*p* < .01) 2. The implementation of screen time restrictions was associated with lower levels of child screen time (*p* < .05) 3. Allowing children to use screens during meals was not associated with child screen time 4. Greater parental self-efficacy to restrict child screen time was associated with lower levels of child screen time (*p* < .05)
74	Thompson et al., 2015	Longitudinal	835	United States	0–4, 2.10 (1.40)	1. Parental Rules (parent reported implementation of content rules) 2. Parenting Style (measure of maternal permissiveness)	Average daily screen time (parent reported)	1. Implementing content rules was associated with higher overall child screen time (*p* < .05) 2. A permissive parenting style was not associated with child screen time
75	Truglio et al., 1996	Longitudinal	271	United States	3–5	Parental Rules (parent reported implementation of child TV time and content limits)	Week-long TV diary (parent reported). Responses were categorised into child and general audience entertainment viewing	The implementation of rules was not associated with child entertainment viewing but was associated with lower levels of general audience entertainment viewing (*p* < .001)
76	Vaala and Hornik, 2014	Cross-sectional	698	United States	0.2–2.25	Parental Modelling (parent self-reported total weekly screen viewing)	Total weekly screen viewing (parent reported)	Higher parental screen time predicted higher levels of child screen time (*p* < .01)
77	Vandebosch & Cleemput, 2007	Cross-sectional	608	Belgium	2.5–6, 4.69	1. Parental Modelling (parent self-reported total weekly screen time) 2. Parental Rules (parent reported restrictive mediation)	Total weekly screen time (parent reported)	1. Higher parental screen time was associated with greater child screen time (*p* < .001) 2. Parent reported use of restrictive mediation was not associated with child screen time
78	Vandewater et al., 2005	Cross-sectional	1065	United States	0–6, 3.20 (1.82)	1. Parental Rules (parent reported implementation of time and content rules) 2. Parental Co-Viewing (parent reported presence during children’s TV use)	Total daily screen time (parent reported)	1. Parental time rules were significantly associated with reduced screen time (*p* < .001) however, content rules were associated with higher screen time (*p* < .001) 2. Parent presence during children’s TV use was associated with higher child screen time (*p* < .001)
79	Vandewater et al., 2007	Cross-sectional	1051	United States	0–4	Parental Rules (parent reported implementation of TV time and content rules	Average daily TV viewing time (parent reported. Responses were dichotomised as > 2 or < 2 h per day to reflect AAP screen recommendations	Time and content rules were not associated with children exceeding the AAP recommendations
80	Varadarajan et al., 2021	Cross-sectional	718	India	0–5	Parental Modelling (parent self-reported average daily screen time. Responses were > 2 or < 2 h of screen use per day)	Week-long screen time diary (parent reported). Responses were dichotomised as > 1 or < 1 h of screen time per day to reflect the WHO recommendations	Higher parental viewing time (> 2 h) was significantly associated with exceeding the recommended screen time hours (> 1 h) in children aged 6–23 months and 24–60 months (*p* < .01 and *p* < .001, respectively)
81	Veldhuis et al., 2014	Cross-sectional	3067	Netherlands	5, 5.80 (0.40)	1. Parental Rules (parent reported implementation of rules about when and how long children could watch TV)	Average daily TV viewing time (parent reported. Responses were dichotomised as > 2 or < 2 h per day to reflect international recommendations	1. The implementation of rules about when and how long children can watch TV predicted children not viewing > 2 h of screen time per day (*p* < .05)
82	Wang et al., 2022	Cross-sectional	1424	China	3–6, 4.52 (0.86)	1. Parental Modelling (parent self-reported home-based screen time per day. Responses were dichotomised as > 1 or < 1 h of screen time per day) 2. Parental Rules (parent reported clarity of screen time rules) 3. Mealtime Viewing (parents reported the number of meals children ate in front of a screen) 4. Parental Co-Viewing (parent reported parent–child viewing behaviour)	Average daily home-based screen time per day (parent reported). Responses were dichotomised as > 1 or < 1 h of screen time per day	1. Mother’s and father’s viewing > 1 h of screen time was significantly associated with children viewing > 1 h of screen time per day (both *p* < .001) 2. Unclear screen time rules were significantly associated with children viewing > 1 h of screen time per day (*p* < .001) 3. Greater frequency of eating in front of a screen was associated with children viewing > 1 h of screen time per day (*p* < .001) 4. Parent–child viewing was associated with children viewing > 1 h of screen time per day (*p* < .001)
83	Warren et al., 2003	Cross-sectional	121	United States	1–5	1. Parental Modelling (parent self-reported total weekly screen time) 2. Parental Rules (parent reported implementation of screen time rules on time and content) 3. Parental Co-Viewing (parent reported co-viewing with their children)	Total weekly screen time (parent reported)	1. Parent screen viewing was associated with higher child screen viewing (*p* < .001) 2. Parent’s implementation of screen time rules was not associated with child screen time 3. Parental co-viewing was not associated with child screen time
84	Wiseman et al., 2019	Cross-sectional	138	Australia	3–5	1. Parental Rules (parent reported implementation of controlling parenting practices on indoor and outdoor play, and screen time) 2. Reinforcement Tool (parent reported use of screen time as a reward or to control behaviour)	Total weekly screen time	1. The implementation of controlling parenting practices was associated with reduced child screen time (*p* < .001) 2. The use of screen time as a reinforcement tool was not associated with child screen time
85	Wu & Ye, 2023	Longitudinal	514	China	Baseline: 3.53 (0.29) Follow-up: 5.50 (0.32)	1. Parental Modelling (mother and father self-reported daily screen time. Categorised as < 1 h, 1–2 h or > 2 h of screen time per day) 2. Parental Rules (parent reported implementation of screen time rules)	Average daily screen time (parent reported). Responses were dichotomised as > 1 or < 1 h of screen time per day	1. Longer screen time of mothers was associated with children exceeding screen time guidelines at both baseline and follow up (*p* < .01 and *p* < .001, respectively). Longer screen time of fathers was associated with children exceeding screen time guidelines at both baseline and follow up (*p* < .01 and *p* < .001, respectively) 2. The absence of clear screen time rules was associated with children exceeding screen time guidelines at baseline and follow up (both *p* < .001)
86	Xu et al., 2014	Cross-sectional	2014	Australia	2, 2.0	1. Parental Modelling (parent self-reported daily screen time. Responses were dichotomised as > 2 or < 2 h per day) 2. Parental Rules (parent reported implementation of program and time rules) 3. Mealtime Viewing (parent reported frequency of mealtime screen viewing) 4. Parental Self-Efficacy (parent self-efficacy for raising an infant)	Average daily screen time. Responses were dichotomised as > 1 or < 1 h of screen time per day	1. Mother’s viewing of > 2 h per day was associated with children viewing > 1 h per day (*p* < .001) 2. Neither program or time rules predicted children viewing > 1 h of screen time per day 3. Higher reported frequency of mealtime viewing was associated with children viewing > 1 h of screen time per day (*p* < .001) 4. Parental self-efficacy did not predict children viewing > 1 h of screen time
87	Yalçin et al., 2002	Cross-sectional	350	Turkey	3–6, 5.20 (0.80)	Parental Modelling (mother and father self-reported average daily screen time)	Average daily screen time (parent reported)	Mothers and fathers screen time was associated with higher child screen time (both *p* < .001)

*AAP* American Academy of Paediatrics, *AU* Australia, *BE* Belgium, *CA* Canada, *DVD* Digital Versatile Disc, *MTSD* Mobile Touch Screen Device, *SK* South Korea, *TechU-Q* Technology Use Questionnaire, *TV* Television, *WHO* World Health Organisation

Where the mean age was not reported, but the upper range value was < 6 years, the mean age was not sought from the authors of the primary studies

### Measurement of Child Screen Use

Following data extraction, two conceptually different measures of screen use were identified; total screen use and adherence to screen time guidelines. Though related, the measures serve different purposes. Total screen use offers an overall estimate of screen-related activities, whereas measures of guideline adherence assess screen use as a function of meeting or exceeding defined time limits. Both measures contribute to a holistic understanding of screen use, however given their conceptual differences they were considered separately. All screen use measures were assessed using parent proxy report.

Sixty-eight studies reported outcomes of total screen use. Of these, 45 studies measured an estimated average daily screen time; 13 measured total weekly screen time; five used screen use recording diaries; two considered the frequency of screen media access and one considered total background TV exposure. In addition, a single study considered the averages of amassed screen time viewing of child and general (e.g. news) TV programmes.

Nineteen studies used dichotomous outcomes of screen time guideline adherence. Of these, nine studies used guidelines equivalent to those suggested by the World Health Organisation (WHO; i.e., > 1 vs. < 1 h); eight used guidelines equivalent to those suggested by the American Academy of Paediatrics (AAP; i.e., > 2 vs. < 2 h); and two dichotomised screen time as high or low according to whether children had > 4 or < 1 h(s) of screen time.

### Impact of Parenting Factors on Screen Use

Table 2 presents a summary of results regarding the association of parenting factors with both child total screen use and child guideline adherence. Importantly, where an overall effect on total screen use or guideline adherence was not available, but subgroup effects were described in a study (e.g. separate effects of parental modelling on total screen use for boys and girls), the subgroup effects were reported independently in the table. An exception was made for studies comparing media types, such that an overall effect was overlooked in favour of reporting separate effects per device type (e.g. separate effects on TV vs. tablet viewing). This was done to explore the role of media type on the relationship between parenting factors and screen use. In total, eight studies (15 effects) measured outcomes of portable media device use. Due to the limited number of available studies considering portable devices, we were unable to identify clear trends or patterns in the data regarding the role of screen media type.

**Table 2:** Associations of parental factors with total child screen use and adherence to screen time guidelines

Parenting factor	Association with children’s total screen use			Strength of Association	Adherence to guidelines			Strength of Association
	Positive	Negative	No association		Non-Adherence	Adherence	No Association	
Parental Knowledge								
Knowledge of health outcomes	**3**	73		IN				
Knowledge of guidelines		47	27	IN				
Parental Modelling	1, 1, 1, 1, **3,** 4, 6, 8, 9, 10, 11, 14, 14, 19, 19, 22, 22, 22, 22, 25, 26, 26, 30, 30^n^, 34, 34, 36, **36**, **36**, 39, 39, 46, 46, 46, 46, 50, **50**, 50, **50**, 51, 56, 57, **60**, 61, 61, **61**, 61, 64, 64, 66, 68, 68, 70, 71, 76, 77, 83, 87, 87	55	2, 27, 57, 59, 64, 70, 71, 71, 71, 72	+/S	7, 24, 38, 42, 46, 46, 80, 80, 82, 82, 85, 85, 85, 85, 86			S
Parenting practices								
Parental Rules	**60**, 74, 78	6, 17, 18, 19, 19, 21, 21, 27, 30, 48, 53, 54, 56, 71, 71, 71, 71, 73, 75, 78, 84	5, 10, 27, **29**, **29**, 30, 59, 66, 75, 77, 83	−/M	7, 40	28, 49	4, 79	U
Mealtime Viewing	15, 18, 23, 23, 41, 71, 71, 72		71, 71, 73	+/M	7, 40, 82, 86			S
Co-Viewing	10, 23, 43, 54, 70, 70, 78	17	23, 23, 23, 30, 30, 83	U	82		40	IN
Reinforcement Tool	16, 16, 23, 23, 58, 71, 71, 71		47, 59, 71, 84	+/M				
Mood Regulation Tool	16, 16, 23, 23, 58, **61**		47, 61, 61, 61	+/M				
Babysitting Tool	9, 23, 23, 58			+/S				
Parental Self-Efficacy	44, 56	11, 13, **13**, 13, 22, 22, 31, 33, 51, 52, 62, 73	17	−/S				
Parenting Style								
Authoritative	**45**	20^r^, 20^ s^, 35^f^, 37, 67^i^, 67^j^	35^ h^, 66	−/M		12		IN
Authoritarian	20^r^, 20^ s^, 35^ h^, 35^f^, 37	**45**	67^i^, 67^j^	+/M	12			IN
Permissive	20^r^, 20^ s^, 35^ h^, 35^f^, 37, **45**		67^i^, 67^j^, 74	+/M	12			IN
Neglectful	37			IN				

Each ID represents an effect from the articles reported in Table 2. Where multiple effects were described in an article, the same article ID has been used. For example, this may be applicable where a study has described separate effects for boys and girls (e.g. Abbott et al., 2016). For a detailed overview of each effect, please refer to Table 2. Bolded effects represent portable media types. Association: Insufficient = (IN). Moderate = (M). No association = (NA). Strong = (S). Unclear = (U). Directionality of effect: Negative association (−). Positive association = ( +)

#### Total Screen Use

Parental modelling was strongly positively associated with total screen use (59/70 positive associations, 84.3%). The implementation of screen use rules was moderately negatively associated with total screen use (21/35 negative associations, 60%). Mealtime viewing (8/11 positive associations, 72.7%), and the use of screens as both a reinforcement (8/12 positive associations, 66.7%) and mood regulation tool (6/10 positive associations, 60%), were moderately positively associated with total screen use. The use of screens as a babysitting tool was strongly positively associated with total screen use (4/4 positive associations, 100%). However, parental co-viewing had an unclear association with total screen use. Parental self-efficacy was strongly negatively associated with total screen use (12/15 negative associations, 80%). An authoritative (6/9 negative associations, 66.7%) parenting style was moderately negatively associated with total screen use, while authoritarian (5/8 positive associations, 62.5%) and permissive (6/9 positive associations, 66.7%) styles were moderately positively associated with total screen use. Insufficient data were available to assess the effect of a neglectful style and parental knowledge of health outcomes and guidelines on total screen use.

#### Screen Time Guideline Adherence

Parental modelling (15/15 non-adherence associations, 100%) and mealtime viewing (4/4 non-adherence associations, 100%) were strongly associated with children exceeding screen time guidelines. The implementation of screen use rules was unclearly associated with children exceeding screen time guidelines (2/6 adherence associations, 33.3%). Insufficient data were available to assess the role of parental co-viewing, and authoritative, authoritarian and neglectful parenting styles with guideline adherence. The impact of parental knowledge, self-efficacy, neglectful parenting styles and the use of screens as a babysitting, mood regulation or reinforcement tool on guideline adherence was not examined in the selected studies.

### Quality Assessment

Study level quality assessment revealed that most studies were of high methodological quality (see supplementary Table 2). Of the included studies, methodological weaknesses (i.e. can’t tell or no responses) were observed in the representativeness of the target population (8.0%), the suitability of exposure and outcomes (20.7%), and the completeness of outcome data (5.7%). Across all studies, it was deemed that they each accounted for confounders in the design and analysis and that the exposure occurred as intended.

## Discussion

The current systematic review offers a comprehensive and current state of literature in relation to the effect of modifiable parenting factors and parenting style on the screen use of children. Moreover, it addresses the demand for a better understanding of parental practices in the context of young children’s screen use (Morawska et al., 2023).

Though we predicted greater screen use knowledge would be associated with less total screen use (H1a), there was insufficient evidence to draw conclusions, highlighting the need for further research in this area. In line with the findings of Kaur et al. (2019), Xu et al. (2015) and Veldman et al. (2023), and supporting our predictions, greater parenting self-efficacy was associated with lower total screen use (H1b). The implementation of screen use rules and their effect on total screen use was subject to mixed findings in previous reviews, with two finding a negative association (Cillero & Jago, 2010; Kaur et al., 2019), and five finding no association (DeCraemer et al., 2012; Duch et al., 2013; Jago et al., 2013a; Veldman et al., 2023; Xu et al., 2015). Our focus on elements of parenting consolidates the findings of previous reviews, offering a robust summary of the evidence. Consistent with our prediction, we found that the implementation of parental screen use rules was associated with lower total screen use (H1c). However, the implementation of rules was unclearly associated with child screen time guideline adherence. This uncertainty may reflect the scarcity of research using guideline adherence outcomes, having only been examined on five occasions, as opposed to the 35 studies that considered total screen use.

Supporting our hypothesis, higher levels of parental modelling were associated with higher total screen use in children (H2a). In addition, we found that higher levels of parental modelling were associated with children exceeding screen time guidelines. These findings are consistent with previous reviews (De Craemer et al., 2012; Duch et al., 2013; Kaur et al., 2019; Veldman et al., 2023; Xu et al., 2015), confirming the important role that parents play in modelling healthy screen use behaviours to their children. Contrary to predictions but consistent with the findings of previous reviews (Jago et al., 2013a; Veldman et al., 2023), co-viewing with parents was unclearly associated with total child screen use (H2b). The inconsistency of these findings points towards the complexity of parental co-viewing. It is likely that the effect of co-viewing is moderated by the intentions of parents. Intentionally co-viewing for the purpose of modelling healthy screen use behaviour or monitoring use may produce significantly different outcomes in child screen use compared to parents’ incidentally co-viewing with their children. Where the former serves to control, guide and scaffold healthy screen use behaviour in children (Sims & Colunga, 2013), the latter operates to model engagement in screen use. As such, consideration of parental intentions while co-viewing is essential to determine its importance moving forward.

In addition to consolidating existing knowledge, our results provided new evidence for factors that have been unobserved in previous reviews. As predicted, higher levels of mealtime viewing were associated with higher levels of total screen use (H2c). We also found that higher levels of mealtime viewing were associated with children exceeding screen time guidelines. As expected, the use of screens as a babysitting (H2d), mood regulation (H2e) and reinforcement tool (H2f), was associated with greater total screen use.

Contrary to the findings of previous reviews (e.g. Jago et al., 2013a; Veldman et al., 2023; Xu et al., 2015), parenting style emerged as an important predictor of screen use. As predicted, an authoritative parenting style was associated with reduced screen use (H3a). This result is unsurprising, as authoritative parents are likely to exert control over their child’s screen use through the implementation of screen use rules (Eastin et al., 2006). However, contrary to predictions, both authoritarian (H3b) and permissive styles (H3c) were associated with higher screen use. The authoritarian parents may be more inclined to use screen use as a reward-based motivator (and the removal of screen use as a punishment), thus inflating children’s overall engagement (Caylan et al., 2021). Moreover, authoritarian parents may also spend less time interacting with their children (e.g. co-viewing), therefore, leading to increased use of screens as a babysitting tool. Permissive parents are low in demandingness and therefore, are less likely to implement screen use rules, leading to higher total screen use (Eastin et al., 2006). Given insufficient evidence, more research is required to determine the relevance of a neglectful parenting style. Notwithstanding, our findings point to the importance of authoritative, authoritarian and permissive parenting styles in child screen use. The relationship, however, is likely mediated by parenting practices such as the implementation of screen use rules.

Overall, our results suggest that parental modelling, self-efficacy and the implementation of screen based rules are important modifiable predictors of screen use in children. Likewise, parenting practices such as allowing children to use screens during mealtimes and the use of screens as a parenting tool for the purpose of mood regulation, reinforcement and babysitting also serve as modifiable targets. These findings support theory indicating the importance of modifiable parenting factors on child screen use. In particular, we highlighted the unique contribution of most of the modifiable factors proposed in the socio-ecological model of Morawska et al. (2023), with the exception of parental knowledge. Finally, we identified that parenting style appears to play an important role in predicting child screen use.

Our findings should be considered in the context of the methodological characteristics of the primary studies. Most studies included in this review (89.7%) were cross-sectional and therefore, were unable to draw causal inferences. As such, there is a great need for more rigorous longitudinal studies that may allow more robust conclusions about the association between parenting factors and screen use.

Though quality assessment indicated that the majority of included studies had appropriate outcome measures, this review highlights many gaps in the assessment of screen use. Strikingly, apart from one (i.e. Truglio et al., 1996), all included studies only considered the time spent using screen devices. As such, there has been a failure to capture important elements of screen use such as the purpose of use and the content being accessed. Morawska et al. (2023) suggest that to accurately understand the influences of child screen use and subsequently design informed interventions, measures must capture the variety, type and extent of screen exposure. This is particularly relevant in light of recent technological advancements in screen media as their utility continues to expand.

Of the included studies, just under half (46%) were conducted in the last 5 years, highlighting the evolving state of screen use literature. In spite of such growth, only eight studies considered screen use in the context of portable screen devices, supporting claims that screen technology is “vastly outpacing research” (Madigan et al., 2020). Vizcaino et al. (2019) emphasise that the relative paucity of studies exploring differences in device type is a critical gap in the literature that may conceal important patterns in screen use behaviour. In the current context, it is possible that different media types may introduce disparities in content, usage patterns and accessibility; potentially impacting the role of parenting factors. In particular, portable devices allow more ready use of screen time as a tool for mood regulation and babysitting, both of which were associated with higher screen use. Furthermore, in addition to the lack of studies assessing the type and content of screen use, we found that screen use was only measured using parent proxy-report. Despite being acknowledged as the most common method of assessment in existing screen use research, such self-report measures suffer from low validity (Boase & Ling, 2013). In fact, several studies have found that there is poor correspondence between objective measures of child screen use and caregiver self-report (Barr et al., 2020; Parker et al., 2022; Radesky et al., 2020). Moreover, these measures are vulnerable to recall and social desirability biases (Duch et al., 2013).

### Strengths and Limitations

Our review had several strengths; most notably, its position as the first review with a concentrated approach on the effect of parenting factors on child screen use. Unlike previous reviews that have considered the influence of parenting in conjunction with a broader array of correlates, our approach provides a nuanced examination of the role of modifiable elements of parenting and parenting style. Thus, we offer a comprehensive foundation from which policymakers, researchers and clinicians can draw from. This review also had the advantage of considering effects separately (e.g. separate effects for boys and girls) in the absence of a total effect on screen use. Such an approach ensured that no important data was lost, and therefore, allowed for a complete exploration of the subject. While subgroup effects were considered, the exploration of these effects was inconsistent across the included studies, therefore preventing direct comparisons. Our search strategy returned a large number of studies for the screening phase, permitting an exhaustive search for relevant literature and minimising the chances of missing critical evidence. The resulting number of primary studies included in this review also serves as a strength. While meta-analysis was not conducted, the volume of studies used enhances the comprehensiveness and robustness of our conclusions.

Nevertheless, our review has many limitations that may impact the validity and relevance of our results. Most pressingly, the heterogeneity of the included studies posed a major limitation, precluding meta-analysis, thus we were not able to quantify the extent to which modifiable parenting factors and parenting style influenced child screen use. Without establishing an overall effect size, the relative importance of each of these factors is unknown. Though narrative synthesis can identify targets for intervention, it is incapable of establishing their priority, as offered through meta-analysis. Sub-group analyses were considered to overcome this limitation however, there was inadequate reporting of necessary statistical information across studies. While a summary coding system was adopted to determine the strength of evidence for each factor, it is acknowledged that this system may introduce sample size bias, where the consistency of findings is prioritised over study quantity. For example, limited but uniform findings (e.g. 4/4 studies supporting a positive relationship between babysitting and screen use) were classified as “strong,” while a larger evidence base with some variability (e.g. 8/11 studies supporting a positive relationship between mealtime viewing and screen use) was only considered “moderate.” Moreover, the preclusion of meta-analysis eliminated the possibility of exploring the moderating role of screen media type on the effect of parenting factors on screen use.

## Implications and Conclusions

The omnipresence of screens in contemporary society has coincided with increased screen use in children (Barber et al., 2017) and child screen is influenced by the home environment, particularly elements of parenting (Pereira et al., 2021; Rhodes et al., 2020). As such, interventions that target parents may be a promising avenue to ensure the responsible consumption of screen use in children. This study has demonstrated that parental modelling; parental self-efficacy; parents’ implementation of screen use rules; mealtime viewing; and parents’ use of screens as a babysitting, mood regulation and reinforcement tool act as modifiable factors on child screen use, supporting the socio-ecological model proposed by Morawska et al. (2023). Therefore, they should be considered as key intervention targets, focussed on educating parents on how each factor influences child screen use and how to incorporate healthy screen use habits into their child’s routine (e.g. appropriate quantity and quality of screen use, implementing rules, and role-modelling healthy behaviours). Given the use of screens as a parenting tool, such an intervention should also look to equip parents with practical (screen-free) approaches to behaviour management to reduce dependency on screens.

While our findings pose an exciting starting point from which intervention can be moulded, they only provide the individual contribution of each of these factors. As such, greater research is required to determine how the identified factors work in combination to influence screen use. In addition, further research is required to determine the role of identified modifiable factors such as parental knowledge and co-viewing. Parental knowledge, of both screen use guidelines and health outcomes, has been subject to limited research and therefore requires greater attention within the literature to establish its importance in child screen use behaviour. Further, establishing parents’ intentions behind co-viewing may clarify its importance. Such research should look to employ longitudinal research methods, to help determine the causality of effects.

Intervention efforts should be considered in the context of the assessment issues surrounding children’s screen use. Current literature has made significant headway in assessing screen use employing time-based metrics, yet these are subject to many limitations. There is a dire need for future research to develop a measure that offers a more nuanced, valid and reliable assessment of screen use. Notwithstanding, the development of appropriate screen use measurement tools is a complex challenge (Byrne et al., 2021; Thompson & Tschann, 2020). We offer the following directions for future research and measurement in this field. First, on top of recording how often screens are used, the studies should look to account for how they are being used. Engagement in entertainment, educational or potentially harmful content while using screens may have different effects on development (Guellai et al., 2022), and may potentially be affected by parenting factors in different ways. Similarly, an understanding of differences across fixed and portable devices may also be potentially important (Vizcaino et al., 2019). Our review has not only revealed future directions for research but has highlighted the important role that parenting factors have on preschool-aged children. The increasing prevalence of screen media is a pressing concern due to its association with negative developmental outcomes therefore, targeting parenting practices may be a promising avenue to mitigate potential adverse effects.

## Supplementary Information

Below is the link to the electronic supplementary material.

Supplementary file1 (DOCX 37 kb)
