# Supplementary material for: The Impact of Modifiable Parenting Factors on the Screen Use of Children Five Years or Younger: A Systematic Review
Source: Clin Child Fam Psychol Rev. 2025 May 1;28(2):458–90. doi: 10.1007/s10567-025-00523-9 (PMC12162779; doi:10.1007/s10567-025-00523-9)
Supplement: Supplementary file 1 — Supplementary file1 (DOCX 37 kb) [file 10567_2025_523_MOESM1_ESM.docx]

**Supplementary Table 1**

*Free-text Words Used in the Search Strategy*

| Child | Child*, preschool*, pre school*, primary school*, infant*, toddler*, |
| --- | --- |
| Parent | Parent*, mother*, father*, maternal, paternal, caregiver |
| Screen Use | Screen use, screen time, screen media, screen exposure, screen sedentary time, television*, computer*, smartphone*, tablet computer*, mobile phone*, mobile media, TV, TV’s, TVs, sedentary behaviour, sedentary behavior, digital media*, electronic media*, electronic device*, media use, digital technolog*, DVD, video game*, electronic game*, screen device*, iPad*, iPod*, iPhone*, computer* |

*Note*. Abbreviations: Digital Versatile Disc (DVD). Television (TV).

Supplementary Table 2. Study Level Quality Assessment

| Study | Are participants representative of  the target population | Are measures appropriate regarding both the outcome and intervention (or exposure) | Are there complete outcome data? | Are the confounders accounted for in the design and analysis? | During the study period, is the  intervention administered (or exposure  occurred) as intended. | Quality Rating (out of 5) |
| --- | --- | --- | --- | --- | --- | --- |
| Abbott et al., 2016 | Yes | Yes | Yes | Yes | Yes | 5 |
| Akbayin et al., 2023 | Yes | Can’t Tell | Yes | Yes | Yes | 4.5 |
| Ali and Alma’aytah, 2022 | Yes | Yes | Yes | Yes | Yes | 5 |
| Asplund et al., 2015 | Yes | Yes | Yes | Yes | Yes | 5 |
| Barr et al., 2010 | Yes | Yes | Yes | Yes | Yes | 5 |
| Barr-Anderson et al., 2011 | Yes | No | Yes | Yes | Yes | 4 |
| Bassul et al., 2021 | Yes | Yes | Yes | Yes | Yes | 5 |
| Bernard et al., 2017 | Yes | No | Yes | Yes | Yes | 4 |
| Beyens and Eggermont, 2014 | Yes | Yes | Yes | Yes | Yes | 5 |
| Bleakley et al., 2013 | Yes | Yes | Yes | Yes | Yes | 5 |
| Carson and Janssen, 2012 | Yes | Yes | Yes | Yes | Yes | 5 |
| Caylan et al., 2021 | Yes | Yes | Yes | Yes | Yes | 5 |
| Chen et al., 2020 | Yes | Yes | Yes | Yes | Yes | 5 |
| Chia et al., 2022 | Yes | Yes | Yes | Yes | Yes | 5 |
| Christakis et al., 2004 | Yes | No | Yes | Yes | Yes | 4 |
| Cingel and Krcmar, 2013 | Yes | Yes | Yes | Yes | Yes | 5 |
| Corkin et al., 2021 | Yes | Yes | Yes | Yes | Yes | 5 |
| Corkin et al., 2022 | Yes | Yes | Yes | Yes | Yes | 5 |
| DeDecker et al., 2015 | Yes | Yes | Yes | Yes | Yes | 5 |
| Detnakarintra et al., 2020 | No | Yes | Yes | Yes | Yes | 4 |
| Downing et al., 2015 | Yes | Yes | Yes | Yes | Yes | 5 |
| Downing et al., 2017 | Yes | Can’t Tell | Yes | Yes | Yes | 4.5 |
| Elias and Sulkin, 2019 | Yes | Yes | Can’t Tell | Yes | Yes | 4.5 |
| Fitzpatrick et al., 2022 | Yes | Yes | Yes | Yes | Yes | 5 |
| Frata et al., 2021 | Yes | Yes | Yes | Yes | Yes | 5 |
| Gao et al., 2022 | Yes | Yes | Yes | Yes | Yes | 5 |
| Goh et al., 2016 | Yes | Yes | Yes | Yes | Yes | 5 |
| Goncalves et al., 2022 | Yes | Yes | Yes | Yes | Yes | 5 |
| Guedes et al., 2020 | Yes | No | Yes | Yes | Yes | 4 |
| Guo et al., 2020 | Yes | Yes | Yes | Yes | Yes | 5 |
| Halpin et al., 2021 | Yes | Yes | Yes | Yes | Yes | 5 |
| Hinkley et al., 2013 | Yes | Yes | Yes | Yes | Yes | 5 |
| Hnatiuk et al., 2015 | Yes | Yes | Yes | Yes | Yes | 5 |
| Holman and Braithaite et, 1982 | Yes | No | Yes | Yes | Yes | 4 |
| Howe et al., 2017 | Yes | Yes | Yes | Yes | Yes | 5 |
| Howie et al., 2020 | No | Yes | Yes | Yes | Yes | 4 |
| Huang et al., 2020 | Yes | Yes | Yes | Yes | Yes | 5 |
| Jago et al., 2013b | Yes | Yes | Yes | Yes | Yes | 5 |
| Jiang et al., 2006 | Yes | Can’t Tell | Yes | Yes | Yes | 4.5 |
| John et al., 2021 | Yes | Can’t Tell | Yes | Yes | Yes | 4.5 |
| Jusiene et al., 2019 | Yes | Yes | Yes | Yes | Yes | 5 |
| Kaur et al., 2022 | Yes | Can’t Tell | Yes | Yes | Yes | 4.5 |
| Kennedy, 2000 | No | Yes | Yes | Yes | Yes | 4 |
| Kieslinger et al., 2020 | Yes | No | Yes | Yes | Yes | 4 |
| Konok et al., 2020 | Yes | Yes | Yes | Yes | Yes | 5 |
| Kourlaba et al., 2009 | Yes | Yes | Yes | Yes | Yes | 5 |
| Lammers et al., 2022 | Yes | Yes | Yes | Yes | Yes | 5 |
| Lampard et al., 2013a | Yes | Yes | Yes | Yes | Yes | 5 |
| Lampard et al., 2013b | Yes | Yes | Yes | Yes | Yes | 5 |
| Lauricella et al., 2015 | Yes | Yes | Yes | Yes | Yes | 5 |
| Lee et al., 2021 | Yes | Yes | Yes | Yes | Yes | 5 |
| Lee et al., 2018 | Yes | Yes | Yes | Yes | Yes | 5 |
| Lee et al., 2009 | Yes | Yes | Yes | Yes | Yes | 5 |
| Levine et al., 2019 | Yes | Yes | Yes | Yes | Yes | 5 |
| Lusted and Joffe, 2018 | Yes | No | Yes | Yes | Yes | 4 |
| Määttä et al., 2017 | Yes | Yes | Yes | Yes | Yes | 5 |
| Morowatsha-  rifabad et al., 2015 | Yes | Yes | Yes | Yes | Yes | 5 |
| Nabi and Krcmar, 2016 | Yes | Yes | Yes | Yes | Yes | 5 |
| Neshteruk et al., 2021 | Yes | Yes | Yes | Yes | Yes | 5 |
| Nevski & Silbak, 2016 | Yes | No | Yes | Yes | Yes | 4 |
| Nikken and Schols, 2015 | Yes | Yes | Yes | Yes | Yes | 5 |
| Njoroge et al., 2013 | Yes | No | Yes | Yes | Yes | 4 |
| Oflu et al., 2021 | Yes | Yes | Yes | Yes | Yes | 5 |
| Pedrotti et al., 2021 | Yes | Yes | Yes | Yes | Yes | 5 |
| Rai et al., 2022 | No | Yes | No | Yes | Yes | 3 |
| Raj et al., 2022 | Yes | Yes | Yes | Yes | Yes | 5 |
| Schary et al., 2012 | No | Yes | Yes | Yes | Yes | 4 |
| Sigmund et al., 2016 | Yes | Yes | No | Yes | Yes | 4 |
| Smith et al., 2010 | Yes | Yes | No | Yes | Yes | 4 |
| Tan et al., 2023 | No | Yes | Yes | Yes | Yes | 4 |
| Tang et al., 2018 | No | Yes | Yes | Yes | Yes | 4 |
| Thompson et al., 2016 | Yes | No | Yes | Yes | Yes | 4 |
| Thompson et al., 2018 | Yes | Yes | No | Yes | Yes | 4 |
| Thompson et al., 2015 | Yes | Yes | Yes | Yes | Yes | 5 |
| Truglio et al., 1996 | Yes | Yes | Yes | Yes | Yes | 5 |
| Vaala and Hornik, 2014 | Yes | Yes | Yes | Yes | Yes | 5 |
| Vandebosch and Cleem-put, 2007 | Yes | Yes | Yes | Yes | Yes | 5 |
| Vandewater et al., 2005 | Yes | No | Yes | Yes | Yes | 4 |
| Vandewater et al., 2007 | Yes | No | Yes | Yes | Yes | 4 |
| Varadarajan et al., 2021 | Yes | Yes | Yes | Yes | Yes | 5 |
| Veldhuis et al., 2014 | Yes | Yes | Yes | Yes | Yes | 5 |
| Wang et al., 2022 | Yes | Yes | Yes | Yes | Yes | 5 |
| Warren et al., 2003 | Yes | Yes | Yes | Yes | Yes | 5 |
| Wiseman et al., 2019 | Yes | Yes | Yes | Yes | Yes | 5 |
| Wu and Ye, 2023 | Yes | Yes | Yes | Yes | Yes | 5 |
| Xu et al., 2014 | Yes | Yes | Yes | Yes | Yes | 5 |
| Yalcin et al., 2002 | Yes | No | Yes | Yes | Yes | 4 |
|  |  |  |  |  |  |  |
